# The paracrine effect of adipose-derived stem cells inhibits osteoarthritis progression

**DOI:** 10.1186/s12891-015-0701-4

**Published:** 2015-09-03

**Authors:** Kazunari Kuroda, Tamon Kabata, Katsuhiro Hayashi, Toru Maeda, Yoshitomo Kajino, Shintaro Iwai, Kenji Fujita, Kazuhiro Hasegawa, Daisuke Inoue, Naotoshi Sugimoto, Hiroyuki Tsuchiya

**Affiliations:** Department of Orthopaedic Surgery, Graduate School of Medical Science, Kanazawa University, 13-1, Takara-machi, Kanazawa, 920-8641 Japan; Department of Physiology, Graduate School of Medical Science, Kanazawa University, Kanazawa, Japan

## Abstract

**Background:**

This study aimed to determine whether intra-articularly injected adipose-derived stem cells (ADSCs) inhibited articular cartilage degeneration during osteoarthritis (OA) development in a rabbit anterior cruciate ligament transection (ACLT) model. The paracrine effects of ADSCs on chondrocytes were investigated using a co-culture system.

**Methods:**

ACLT was performed on both knee joints of 12 rabbits. ADSCs were isolated from the subcutaneous adipose tissue. ADSCs with hyaluronic acid were intra-articularly injected into the left knee, and hyaluronic acid was injected into the right knee. The knees were compared macroscopically, histologically, and immunohistochemically at 8 and 12 weeks. In addition, cell viability was determined using co-culture system of ADSCs and chondrocytes.

**Results:**

Macroscopically, osteoarthritis progression was milder in the ADSC-treated knees than in the control knees 8 weeks after ACLT. Histologically, control knees showed obvious erosions in both the medial and lateral condyles at 8 weeks, while cartilage was predominantly retained in the ADSC-treated knees. At 12 weeks, the ADSC-treated knees showed a slight suppression of cartilage degeneration, unlike the control knees.

Immunohistochemically, MMP-13 expression was less in the ADSC-treated cartilage than in the control knees. The cell viability of chondrocytes co-cultured with ADSCs was higher than that of chondrocytes cultured alone. TNF-alpha-induced apoptotic stimulation was similar between the two groups.

**Conclusions:**

Intra-articularly injected ADSCs inhibited cartilage degeneration progression by homing to the synovium and secreting a liquid factor having chondro-protective effects such as chondrocyte proliferation and cartilage matrix protection.

## Background

Osteoarthritis (OA) is characterized by the destruction of the extracellular matrix and the loss of chondrocyte function [1, 2]. Although OA affects a large proportion of the population there are few, if any, effective therapies available today that alter the pathobiologic course of the disease [3]. For many years, scientists have been searching for ways to inhibit the process of the disease and slow the progression of joint damage [4]. This is the driving force behind numerous ongoing efforts to develop new tissue engineering-based strategies for the treatment of OA [5, 6].

Mesenchymal stem cells (MSCs), with the capacity to differentiate into a variety of cells including osteoblast, tendon, muscle, adipose tissue, and chondrocyte [7, 8], are used in cellular therapy for a broad spectrum of diseases [9]. In a degenerative disease such as OA, the stem cells are exhausted and their capability for proliferation and differentiation decreases [10]. Systemic or local stem cell treatment can promote repair of the articular cartilage [8], but studies have increasingly demonstrated the low survival rate of the transplanted stem cells [11], so recent research has focused on treatments involving the paracrine effect of stem cells. Although stem cells are primarily thought to act through tissue differentiation, they can also act in a paracrine manner via the production of soluble anti-inflammatory factors [8, 12, 13].

In this study, we investigated the clinical application of one type of stem cells, adiposed-derived stem cells (ADSCs), in various fields such as bones, peripheral nerves, menisci, and ligaments [14–16]. ADSCs are safe; they can easily be isolated from subcutaneous adipose tissue and cultured in large quantities, making them a good source of tissue stem cells. Some reports have described the efficacy of an intra-articular injection of ADSCs in animal OA models. [3, 8, 12, 17, 18]. However, the role of ADSCs in the treatment of osteoarthritis is not clear [7, 9, 12, 19].

The primary purpose of this study was to determine whether intra-articularly injected ADSCs prevented or slowed articular cartilage degeneration during osteoarthritis (OA) development in a rabbit anterior cruciate ligament transection (ACLT) model; in particular we focused on the paracrine effect of ADSCs. Secondarily, the mechanism underlying the paracrine effects of ADSCs on chondrocytes was investigated in vitro.

## Methods

The experimental protocol, using an animal model, was approved by the Institute for Experimental Animals, Kanazawa University Advanced Science Research Center. Surgery was performed in accordance with the Guide for the Care and Use of Laboratory Animals published by the US National Institutes of Health (NIH publication no.85-23, revised 1996), and using aseptic techniques.

### Animals

Skeletally mature female Japanese white rabbits, weighing between 2.5 and 3.0 kg, were used for this experiment.

### Adipose-derived stem cells

ADSCs were isolated by modifying a previously reported method [14, 20]. Adipose tissue (1.5 g) was harvested from the posterior cervical subcutaneous adipose tissue of a rabbit and washed with phosphate-buffered saline (PBS) (Wako, Osaka, Japan). The tissue was cut into strips over a period of 5 min. Collagenese (Wako) was dissolved in PBS so that its concentration would be 0.12 % in 25 ml, and was used to digest adipose tissue at 37 °C for 45 min in a water bath. The mixture was shaken every 15 min during the digestion period. Immediately after the reaction was completed, 25 ml of Dulbecco’s modified Eagle’s medium, DMEM (Wako), was added to neutralize collagenase activity. The resulting solution was filtered. The filtrate was centrifuged at 1300 rpm for 6 min at 25 °C, and the supernatant was removed. Next, a pallet of ADSCs was seeded at 5 × 10^4^ cells/cm^2^ in 60.1-cm^2^ tissue culture dishes and cultured with complete medium, DMEM containing 10 % fetal bovine serum (FBS), 100 units/ml penicillin, and 100 μg/ml streptomycin. After being cultured for 1 week, the cells were harvested with 0.25 % trypsin-EDTA, centrifuged at 1300 rpm for 6 min, and washed twice with PBS. The obtained cells were used for intra-articular injections in vivo and co-culture assays in vitro.

#### In vivo experiments

### Induction of experimental OA

Twelve rabbits were used for the *in vivo* experiments. All were anesthetized by intramuscular injection of ketamine hydrochloride (35 mg/kg body weight; Sankyo Pharmaceutical, Tokyo, Japan) and xylazine (5 mg/kg body weight; Bayer, Tokyo, Japan), and intravenous injection of pentobarbital sodium (40–50 mg/kg body weight; Abbott Laboratories, Chicago, IL, USA).

Traumatic degeneration was induced as previously described for the anterior cruciate ligament model [21, 22]. This model is characterized by OA-like damage. Hayashi et al. noted that rabbits show considerable individual variability in osteoarthritis progression at 4 weeks after ACLT [23]. As they suggested, we used matched-pair analysis to examine the paracrine effect of ADSCs on osteoarthritis progression in a stricter manner.

Both knees were shaved and disinfected with iodine. ACLT was performed as described by Yoshioka et al. [22]. Briefly, a medial parapatellar incision was made and an arthrotomy was performed. The patella was dislocated laterally and the knee placed in full flexion. The ACL was visualized and transected with a No.11 blade. An anterior drawing test was performed gently to confirm that the ACL was transected completely. The joint was irrigated with sterile saline and closed. A same operation was performed in the contralateral knee. The knee was opened and the patella was dislocated. After gently performing the anterior drawing test, the joint was irrigated and closed. After the operation, free activity was allowed in the cage without immobilization.

### Injection of ADSCs

ADSCs were isolated from the subcutaneous adipose tissue 3 weeks after ACLT and cultured for 1 week as described above.

For matched –pair analysis of ADSCs, 12 rabbits (24 knees) were used. At 4, 5, 6 weeks after ACLT, ADSCs (2.0 × 10^6^ cells) in 100 μl PBS and 0.1 mL hyaluronic acid (Synvisc®; kindly provided by Teijin Limited, Japan) were intra-articularly injected into the left knee, using a 23 gauge needle. In right knees, the same volume of plain PBS and and hyaluronic acid were injected as a control. Immediately after injection, the rabbit was kept down for 5 min so that injected ADSCs could be attached around the synovium.

Femoral condyles from both knees were harvested at 8 and 12 weeks after surgery following the intravenous injection of 6 ml sodium pentobarbital.

### Macroscopic analysis

The femoral condyles were dissected and stained with India ink (American MasterTech, CA, USA). Macroscopic pictures were taken using a Panasonic LUMIX digital camera (Panasonic, Tokyo, Japan). Gross findings were classified and scored as described in our previous reports [4, 23, 24]. The medial and lateral femoral condyles were individually scored from grades 0 to 5 and the two scores were summed to obtain a cumulative macroscopic osteoarthritis score. A blind assessment was then independently conducted by two individual examiners (KK, TK) and the scores from the two examiners were averaged to obtain an overall score.

### Histological analysis

The dissected distal femurs were fixed in a 4 % paraformaldehyde solution after gross morphological examination. The specimens were decalcified in 4 % EDTA solution, dehydrated with a gradient ethanol series, and embedded in paraffin blocks.

Ten coronal sections were prepared in the coronal plane through the middle of the femoral condyles, and one section from each sample, which included the most severely degenerated area, was used for each of the histological analyses.

The specimens were stained with safranin O or H&E using standard procedures.

Histological sections were visualized using a fluorescence microscope (Keyence Japan, Osaka, Japan), assessed in a blind manner by two individual examiners (TK, KK), and quantified according to the cartilage OA histopathology grading system methodology of the Osteoarthritis Research Society International (OARSI) [25].

### Immunohistochemical analysis

Immunohistochemical examinations were performed as follows. In brief, after deparaffinization, sections were incubated with 0.3 % hydrogen peroxide for 30 min. Then, sections were treated with hyaluronidase for 60 min after which they incubated with mouse anti-human MMP-13 monoclonal antibody (1:20; AnaSpec Inc., San Jose, Ca, USA), or mouse anti-rabbit MMP-3 monoclonal antibody (1:50; Daiichi Fine Chemical Co. Toyama, Japan) [26]. All antibody dilutions were made in PBS. After an overnight reaction with the primary antibody at 4 °C, sections were incubated with labeled polymer-HRP anti-mouse IgG (Dako, Tokyo, Japan) at room temperature for 30 min. Signals were visualized with 3, 3’-diaminobenzidine tetrahydrochloride, and nuclei were counterstained with hematoxylin. A semi-quantitative method that assigns immunohistochemistry values as a percentage of positive cells (MMP-13, MMP-13) was provided for a complete assessment of protein expression, with a maximum score of 100 % [12].

Results of the histological and immunohistochemical analyses were subjected to a blind evaluation by two observers (TK, KK).

### DiI labeling

On the day of intra-articular injection, ADSCs were labeled for cell tracking with a fluorescent lipophilic tracer 1,10-dioctadecyl-3,3,30,30-tetramethylindocarbocyanine perchlorate (DiI; MolecularProbes). For labeling, the cells were re-suspended at 1 × 10^6^ cells/ml in aMEM, and DiI was added at 5 ml/ml in a serum-free DMEM. After incubation for 20 min at 37 °C with 5 % humidified CO2, the cells were centrifuged at 1300 rpm for 6 min and washed twice with PBS, then re-suspended in PBS for the injection.

An examination was performed to determine where the ADSCs existed after the intra-articular injection.

A fresh frozen coronal section containing a central portion of the knee joint was prepared using Kawamoto’s method [27].

#### in vitro

### Primary chondrocyte culture

Primary culture of chondrocytes was performed using articular cartilage tissues harvested from a rabbit. Briefly, thinly sliced cartilage tissues were incubated with 0.15 trypsin with 0.1 % collagenase for one hour. Subsequently, cartilage tissues were incubated in DMEM with 0.15 % collagenase for 2 h 30 min. The cells were then collected by centrifugation, seeded into collagen I coated 24-well plates (collagenI multiwell plates, BD Falcon™), and cultured with DMEM containing 10 % FBS supplemented with 100 units/ml penicillin and 100 mg/ml streptomycin at 37 °C in a humidified atmosphere of 5 % CO2/95 % air. Chondrocytes were grown in monolayer cultures, and were passaged when reaching confluence. Cells at the second passage were used for the assay.

### Co-culture assay

**Fig. 1:**
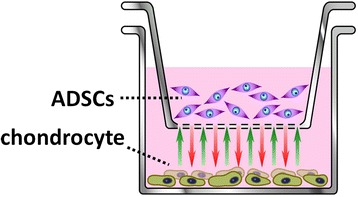
The co-culture system is comprised of ADSCs in Cell culture inserts and chondrocytes in twelve-well plates. ADSCs in itself cannot pass cell culture inserts, but but secreted factors from ADSCs can pass cell inserts and affect chondrocytes

To confirm the paracrine effect of ADSCs for chondrocyte, we used the co-culture system (Fig. 1).

The co-culture groups were established between ADSCs (5 × 10^3^cells/well) in Cell culture inserts (Cell Culture inserts, 0.4 μm pores, BD Falcon™) and chondrocytes (5 × 10^3^cells/well) in twelve-well plates (Cell Culture Insert Companion Plates, BD Falcon™) in DMEM. The control group was established between no cells in Cell culture inserts and chondrocytes only in twelve-well plates (5 × 10^3^cells/well) in DMEM.

After 48 h, cell viability of the chondrocyte group was determined using the MTT assay and compared to that of the co-culture group.

Subsequently, to confirm the paracrine effect of ADSCs for chondrocyte under TNF-α, the culture experiments were divided into 3 groups: chondrocytes (control); chondrocytes with TNF-α (10 ng/mL) (TNF-α); and co-culture of ADSCs and chondrocytes with TNF-α (10 ng/mL) (ADSCs). After 48 h, cell viability of the chondrocytes was determined using the MTT assay and compared among the three groups.

Next, 72 h after TNF-α stimulation, the concentration of MMP-13 in the three groups was measured by ELIZA. The Human Biotrak MMP-13 ELIZA kit (GE Healthcare) was used because these kits were previously shown to be suitable for assaying rabbit MMPs [28, 29].

We believe that quantifying the cellular survival rate enabled us to evaluate the chondrocyte proliferative effects of the paracrine effect of ADSCs, while the MMP-13 quantification allowed us to evaluate the chondrocyte matrix protection effects. TNF-α stimulation induced chondrocyte apoptosis and reproduced the clinical condition of OA.

### Statistical analysis

Statistical analyses were performed using SPSS ver.19.0 (SPSS, Inc, Chicago, Ill). The results are shown as the mean ± standard deviation (SD). The unpaired *t*-test was used. In all analyses, *P* < .05 indicates statistical significance.

## Results

### In vivo

#### Macroscopic analysis

At 8 weeks after ACLT, severe erosion in both medial and lateral femoral condyles was observed in the control group; 75 % of the condyles were graded “erosion”. At the same time, in the ADSC group, near normal cartilage or fibrillation was observed; only 25 % of the condyles were graded “erosion”. In addition, the areas of damaged cartilage appeared to be smaller than those in the control knees in each rabbit.

**Fig. 2:**
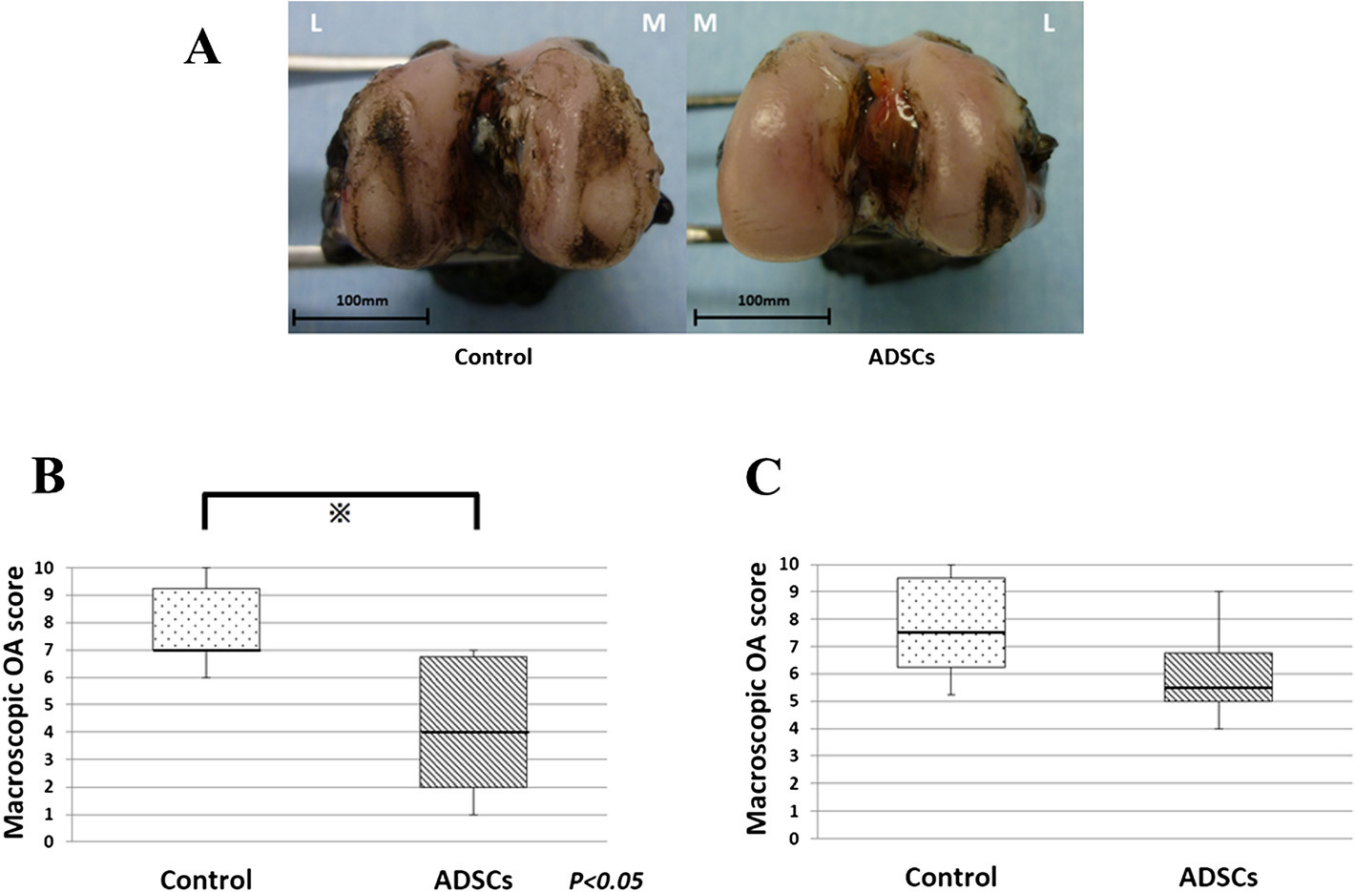
Macroscopic analyses of the femoral condyles. **a** Representative specimen 8 weeks after ACLT. To remove individual viability, both sides of the knees of the same individuals are shown. The surface of the cartilage was stained with India ink to identify any fibrillation and erosion. Laterality is shown as medial (M) and lateral (L). **b** Macroscopic osteoarthritis score 8 weeks after ACLT (n = 6). **c** Macroscopic osteoarthritis score 12 weeks after ACLT (*n* = 6)

Figure 2a shows an example of a typical control and ADSC-treated condyle, 8 weeks after ACLT, stained with india ink. In the control knees, erosions in both the medial and lateral condyles can be seen. On the other hand, in the ADSC-treated knees, no erosion was observed; there were no macroscopically identifiable cartilage lesions in the medial condyle, and little fibrillation in the lateral condyle.

The mean macroscopic OA score (8 weeks) for the control group was 8.2 ± 1.5 while the ADSC group had a mean score of 4.7 ± 2.8 (Fig. 2b); the lower score for the ADSC group indicates less damage to the cartilage surface. This was a statistically significant difference (*P* = 0.019).

At 12 weeks after ACLT, severe erosion was observed in both groups. The mean macroscopic OA score for the control group was 7.5 ± 2.3 while the ADSC group had a mean score of 5.7 ± 2.3 (Fig. 2c). There was no statistically significant difference (*P* = 0.20).

#### Histological analysis

Histologically, 8 weeks after ACLT control knees showed an obvious loss of cartilage in both the medial and lateral condyles, while the cartilage matrix was predominantly retained in the ADSC-treated knees.

**Fig. 3:**
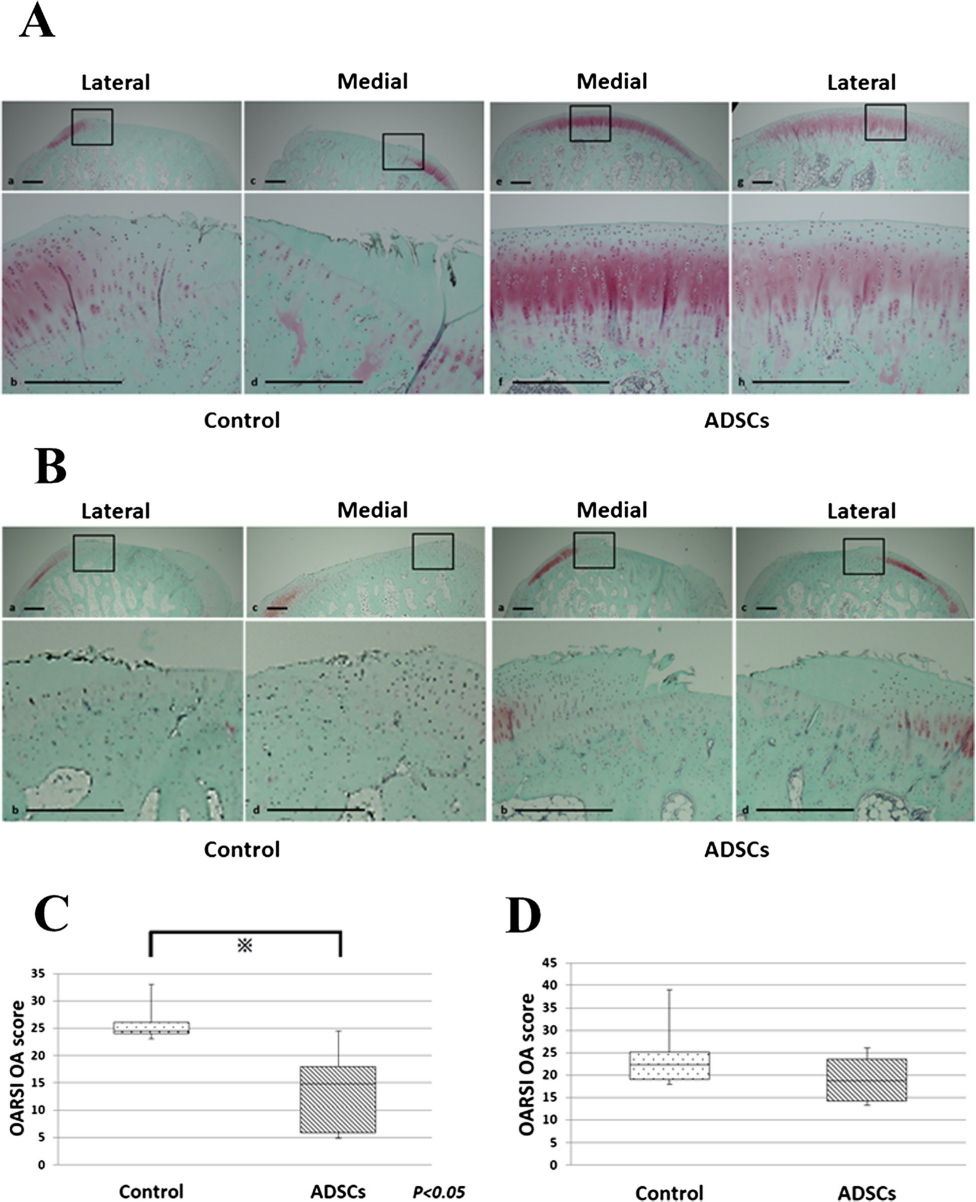
Histological analyses of the femoral condyles. **a** Representative specimen 8 weeks after ACLT. **b** Representative specimen 12 weeks after ACLT. Bothe sides of the knees from same individuals are shown. Distal femur was sectioned coronally and stained with safranin-O. Bars, 500 μm. **c** The OARSI OA score 8 weeks after ACLT (*n* = 6). **d** The OARSI OA score 12 weeks after ACLT (*n* = 6)

Figure 3a shows a typical histological section from a control group condyle and an ADST-treated condyle. The control group sections showed more cartilage defects.

At 8 weeks after ACLT, the mean OARSI score for the control group was 13.0 ± 8.9 while the ADSCs group had a mean score of 25.6 ± 4.1 (Fig. 3c). This was a statistically significant difference (*P* = 0.016).

At 12 weeks after ACLT, greater loss of cartilage was observed in both the control and ADSC groups (Fig. 3b). The mean OARSI score for the control group was 20.0 ± 2.8 while the ADSC group had a mean score of 19.0 ± 7.1 (Fig. 3d). There was no statistically significant difference between the two (*P* > 0.05).

#### Immunohistochemical analysis

MMP-3 and MMP-13 are major proteases degrading the extracellular matrix. The expression of these enzymes was analyzed by immunohistochemistry using samples prepared 8 weeks after ACLT.

For MMP-3, there were no significant differences between the number of positive cells in the ADSC and control groups (data are not shown).

**Fig. 4:**
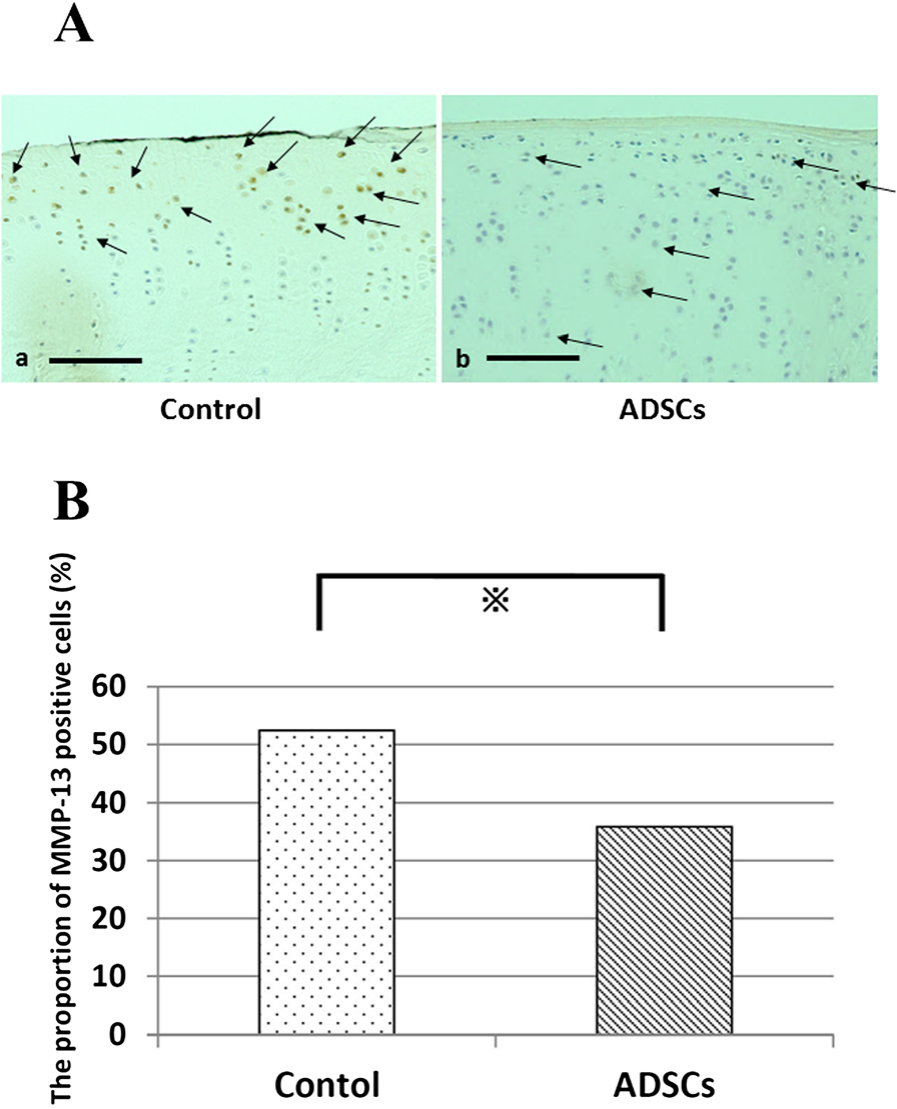
Immunohistochemical analysis for MMP-13 in cartilage. **a** Representive specimens 8 weeks after ACLT, evaluated for MMP-13 in medial femoral condyle. Medial sides of the knees from same individuals are shown. MMP-13 positive cells are indicated by arrows. Bars, 500 μm. **b** The proportion of MMP-13 positive cells (*n* = 6)

The proportion of MMP-13-positive cells, however, was significantly lower in sections of the ADSC groups than in sections of the control group (Fig. 4b).

#### DiI labeling

**Fig. 5:**
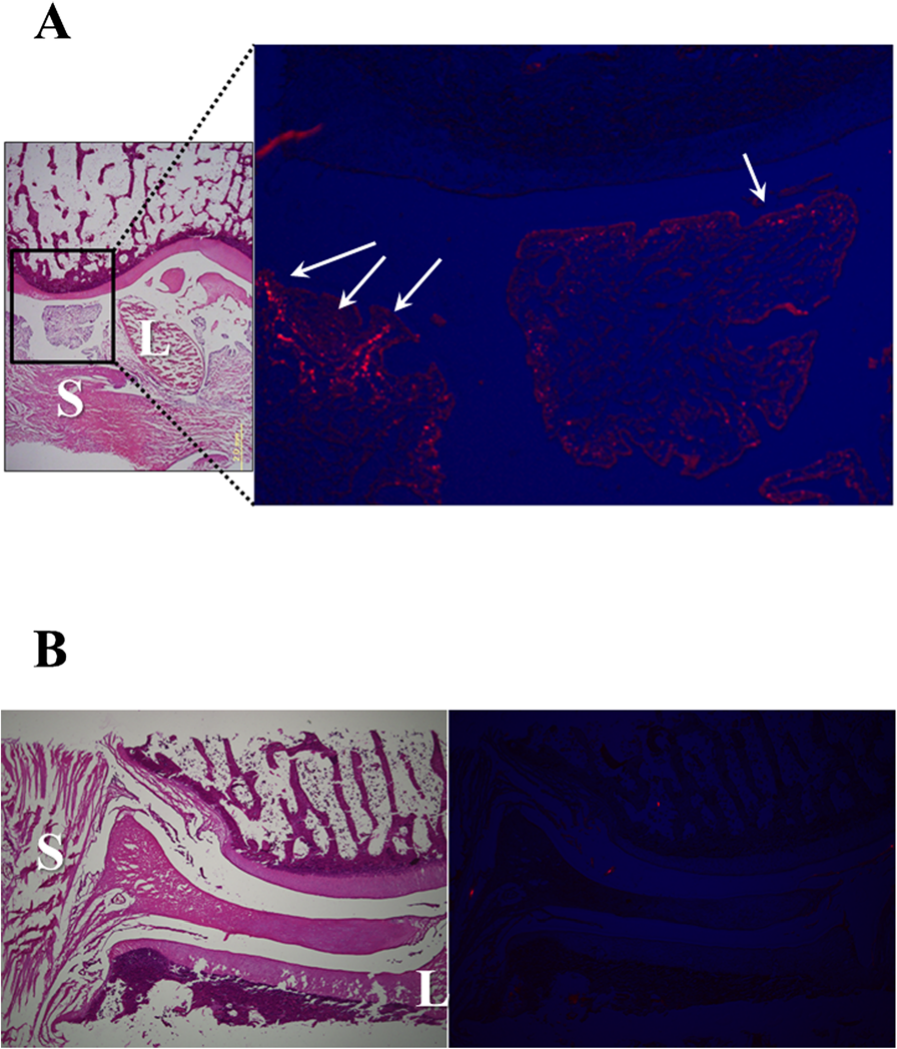
DiI labeling at 8 weeks (**a**) and 12 weeks (**b**) after ACLT; After intra-articular injection of ADSCs labeled with DiI dye, frozen sections were prepared and then stained with hematoxylin and eosin. DiI positive cells survived and homed to the subintimal layers of the synovium (*S*) and ligament (*L*), not in the cartilage at 8 weeks after ACLT

DiI positive areas are shown in Fig. 5a and b. Figure 5a reveals that intra-articular injected cells survived and homed to the subintimal layers of the synovium and ligament, but not the cartilage, 8 weeks after ACLT. On the other hand, at 12 weeks after ACLT, no DiI positive cells were observed (Fig. 5b).

### in vitro

#### Co-culture assay

To confirm the paracrine effect of ADSCs on chondrocytes, their cell viability was evaluated using a co-culture system (Fig. 5).

**Fig. 6:**
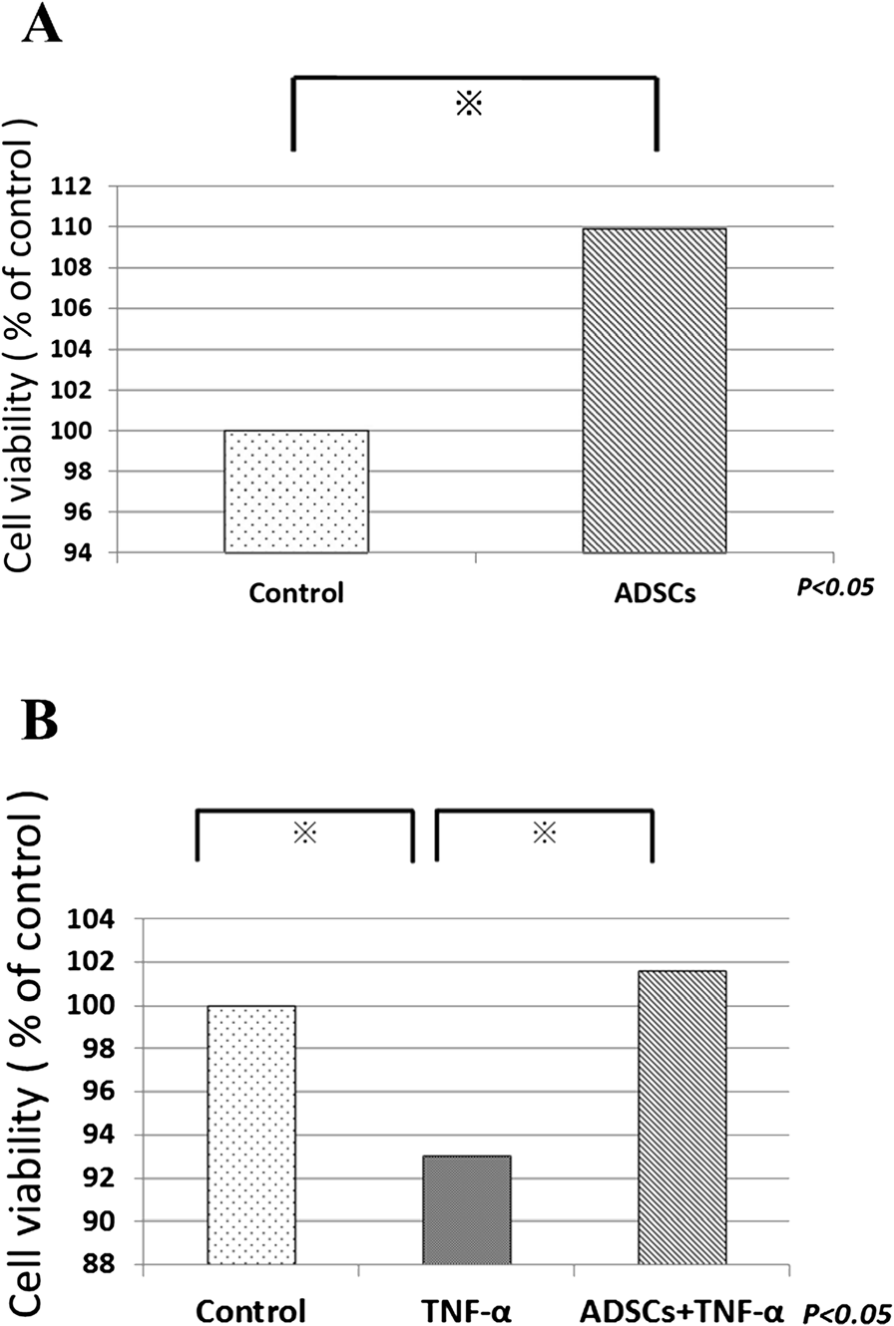
Cell viability of chondrocyte was determined using the MTT assay and compared (**a**) between chondrocyte group and co-culture group, (**b**) between each groups; chondrocytes (control), chondrocytes with TNF-α (10 ng/mL) (TNF-α); and co-culture of ADSCs and chondrocytes with TNF-α (10 ng/mL) (ADSCs + TNF-α)

The cell viability of chondrocytes co-cultured with ADSCs was higher than that of chondrocytes cultured alone (Fig. 6a). Similar results were obtained under TNF-α stimulation. The cell viability of chondrocytes co-cultured with ADSCs was higher than that of those cultured alone under TNF-α stimulation (Fig. 6b).

**Fig. 7:**
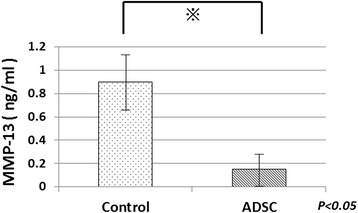
MMP-13 levels in medium of co-culture of ADSCs and chondrocytes with TNF-α and chondrocyte cultured alone with TNF-α. MMP-13 was measured by ELIZA

To confirm the paracrine effect of ADSCs on MMP-13 expression, the concentration of MMP-13 in the co-culture and control groups was evaluated by ELIZA. At 72 h after TNF-α stimulation, the concentration of MMP-13 in the medium of the co-culture group was lower than that in the medium of the chondrocyte alone group, as shown in Fig. 7. This indicates that the expression of MMP-13 was reduced by the paracrine effect of the ADSCs.

## Discussion

In this study, we found that intra-articularly injected ADSCs inhibited cartilage degeneration progression in a rabbit OA model, and homed to intra-articular soft tissue (the subintimal layers of the synovium, ligament). Furthermore, we focused on the paracrine effects of ADSCs; intra-articularly injected ADSCs could secrete a liquid factor having chondro-protective effects, such as chondrocyte proliferation and cartilage matrix protection.

Adipose tissue is known to play a key role in energy balance and metabolic disorders [9]. Moreover, a recent study demonstrated that adipose tissue hosts multipotent stem cells [30]. These cells, called adipose tissue-derived stromal cells or adipose stem cells (ASCs), were first reported in 2001 by Zuk et al. [20]. ADSCs have the capacity to differentiate into a variety of cells, including adipocytes, osteoblasts, chondrocytes, and myocytes under specific culture conditions in vitro [31].

On the other hand, some reports note that when the chondrogenic potential of MSCs derived from bone marrow and from adipose tissue were compared in vitro and in vivo, chondrogenesis was superior in those derived from bone marrow [32, 33].

ADSCs are used for chondro-protective therapy because autologous ADSCs can easily be isolated in large amounts from subcutaneous adipose tissue [14]. Also ADSCs can be harvested less invasively than BMSCs, and many more stem cells can be harvested at one time [34, 35]. In clinical applications, autologous ADSCs should be used from the perspective of safety, and ADSCs should be minimally manipulated. This is why ADSCs are emerging as an alternative to BMSCs in the orthopaedic field [17].

We also focused on the paracrine effect of ADSCs as a new method for chondro-protective therapy.

It is generally believed that the primary therapeutic effect of stem cells occurs through tissue differentiation because stem cells can differentiate toward various types of cells [36]. Lee et al. reported that injected MSCs were present in neocartilage [37]. On the other hand, bioactive factors secreted by stem cells can influence the local tissue environment and exert a protective effect. [38, 39]. Arnold Caplan first proposed MSCs as a trophic mediator for tissue repair [38]. In addition, MSCs have a trophic, anti-inflammatory and immunosuppressive action, by modulating T and B cells and inducing the expression of anti –inflammatory factors, such as interleukin 10 (IL-10), IL-1 receptor antagonist (IL-1 RA), or prostaglandin E2 (PGE2) [17].

In this study, intra-articularly injected DiI-ADSCs homed to intra-articular soft tissue (the subintimal layers of the synovium, ligament) 8 weeks after ACLT, but there were few in articular cartilage. The above results indicate that the paracrine effect of ADSCs which homed in intra-articular soft tissue contributed largely to the inhibition of cartilage degeneration progression, not regeneration by ADSC differentiation.

There have been many reports concerning the therapeutic properties of ADSCs for cartilage defects [40, 41]. In recent years, several researchers have suggested the effectiveness of ADSCs as treatment for OA [7–9, 12, 17, 19, 42].

In our study, osteoarthritis progression was obviously milder in the ADSC-treated knees than in the control knees 8 weeks after ACLT in both macroscopic and histological evaluation. This is the data supporting the efficacy of ADSCs for OA treatment that other reports also have suggested.

Despite the results showing the chondro-protective effects of ADSCs, it is important to mention that the mechanism by which ADSC therapy facilitates tissue repair is still unclear. Toghraie et al. indicated that the injected ADSCs may regenerate degenerative tissue after directly filling the lesion [7], but there is insufficient evidence for this. Other reports have suggested that that injected ADSCs indirectly stimulate the secretion of bioactive factors such as cytokines and growth factors [9, 12, 42, 43]. We also focused on the paracrine effects of ADSCs, especially the effect on chondrocytes. We investigated these paracrine effects using a co-culture system.

Wu et al. investigated the paracrine effect of MSCs on chondrocytes using a co-culture system [44]. Tsuchiya et al. first reported that co-culture of BMSCs and articular chondrocytes enhanced matrix production [45]. Meanwhile, Hildner et al. also reported increased cartilage formation in the co-culture of chondrocytes with ADSCs [46].

Our data showed a significant increase of chondrocytes in co-culture with ADSCs.　Furthermore, although TNF-α led to the death of chondrocytes (Fig. 6b Control VS TNF-α), the effect of TNF-α was suppressed when chondrocytes were co-cultured with ADSCs (Fig. 6b TNF-α VS ADSCs + TNF-α). These results indicate that the paracrine effects of ADSCs have effects such as regulating chondrocyte viability in osteoarthritis.

It is believed that MMP13 causes damage to cartilage in OA, and that this is mediated by chondrocytes in an autocrine or paracrine manner [47]. We showed that production of MMP-13 in articular chondrocytes was reduced when they are treated with injected ADSCs in vivo, and the MMP-13 concentration of culture fluid was also reduced when co-cultured with ADSCs in vitro. These data suggest that ADSCs protect the articular cartilage from degeneration by inhibiting MMP13 expression and secreting multiple growth factors.

In our results, intra-articularly injected ADSCs had an inhibiting effect on cartilage degeneration progression 8 weeks after ACLT, but did not show a significant effect at 12 weeks. Furthermore, no DiI positive cells were observed 12 weeks after ACLT. This is why intra-articularly injected ADSCs might not survive in a joint, so the paracrine effect lasted only weeks.

## Conclusions

Intra-articularly injected ADSCs inhibited cartilage degeneration progression in a rabbit OA model by homing to the synovium and secreting a liquid factor with chondro-protective effects such as regulating chondrocyte viability and cartilage matrix protection.

## Abbreviations

ACLT: Anterior cruciate ligament transection
ADSCs: Adipose-derived stem cells
DMEM: Dulbecco’s modified Eagle’s medium
FBS: Fetal bovine serum
H&E: Hematoxylin & eosin
IL: Interleukin
MMP: Matrix metalloproteinase
MSCs: Mesenchymal stem cells
OA: Osteoarthritis
PBS: Phosphate-buffered saline
PG: Prostaglandin
SD: Standard deviation
TNF-α: Tumor necrosis factor
